# CART cells are prone to Fas- and DR5-mediated cell death

**DOI:** 10.1186/s40425-018-0385-z

**Published:** 2018-07-13

**Authors:** Benjamin O. Tschumi, Nina Dumauthioz, Bastien Marti, Lianjun Zhang, Pascal Schneider, Jean-Pierre Mach, Pedro Romero, Alena Donda

**Affiliations:** 1 Translational Tumor Immunology Group, Department of Fundamental Oncology, Lausanne, Switzerland; 2 Department of Biochemistry, Faculty of Biology and Medicine, University of Lausanne, Lausanne, Switzerland

**Keywords:** CAR T cells, CD8 T lymphocytes, Fas, FasL, Annexin V, *Listeria monocytogenes*, HER2, 4-1BB, CD28, CD3ζ

## Abstract

Adoptive transfer of T cells transduced with Chimeric Antigen Receptors (CAR) are now FDA-approved for the treatment of B-cell malignancies. Yet, the functionality of the endogenous TCR in CART cells has not been fully assessed. Here, we demonstrate that CART cells progressively upregulate Fas, FasL, DR5 and TRAIL, which result in their programmed cell death, independently of antigen-mediated TCR or CAR activation. CART cell apoptosis occurs even when the CAR contains a single (co-)activatory domain such as CD3ζ, CD28 or 4-1BB. Importantly, the dominant role of the Fas and DR5 pathways in CART cell apoptosis is demonstrated by the significant rescue of CART cells upon in vivo blockade by combined Fas-Fc and DR5-Fc recombinant proteins. These observations are of crucial importance for the long-term persistence of CART cells and for the development of new applications including the combined TCR and CAR activation against solid tumors.

**Electronic supplementary material:**

The online version of this article (10.1186/s40425-018-0385-z) contains supplementary material, which is available to authorized users.

## Introduction

Adoptive T cell immunotherapies involving CART cells show impressive clinical responses in advanced cancer [1–5]. Two CART cell products were recently FDA-approved for the treatment of B cell malignancies, and ongoing research aims to extend this approach for the treatment of acute myeloid leukemia [6], multiple myeloma [7] and solid tumors [8, 9]. CART cell therapy applied to solid tumors is facing additional challenges, such as multiple mechanisms of tumor escape, including the highly immunosuppressive tumor microenvironment, and the selection of tumor-specific antigens causing minimal off- and on-target toxicity. Around forty clinical trials are currently open against a large variety of solid malignancies. In contrast to hematological malignancies, a few completed clinical studies showed a good safety profile, but unfortunately, no signs of clinical activity [10]. With the aim to enhance the effectiveness of CART cell therapy, we and others have evaluated the transduction of a CAR into tumor antigen-specific T cells, so as to attack the tumor via combined TCR and CAR activation. However, all in vivo pre-clinical studies have failed so far to demonstrate a combined TCR/CAR activation associated with synergistic antitumor effect [11–14]. In this regard, we have evaluated the functionality of the resident TCR in CART cells, either by using a bacterial infection model or in therapeutic tumor settings. Unexpectedly, we found that independently of TCR activation, CART cells undergo programmed cell death (PCD) associated with the up regulation of Fas, FasL, DR5 and TRAIL expression, resembling the process of Activation Induced Cell Death (AICD), which occurs upon excessive T cell stimulation or during the T cell contraction phase.

## Results and discussion

**Fig. 1:**
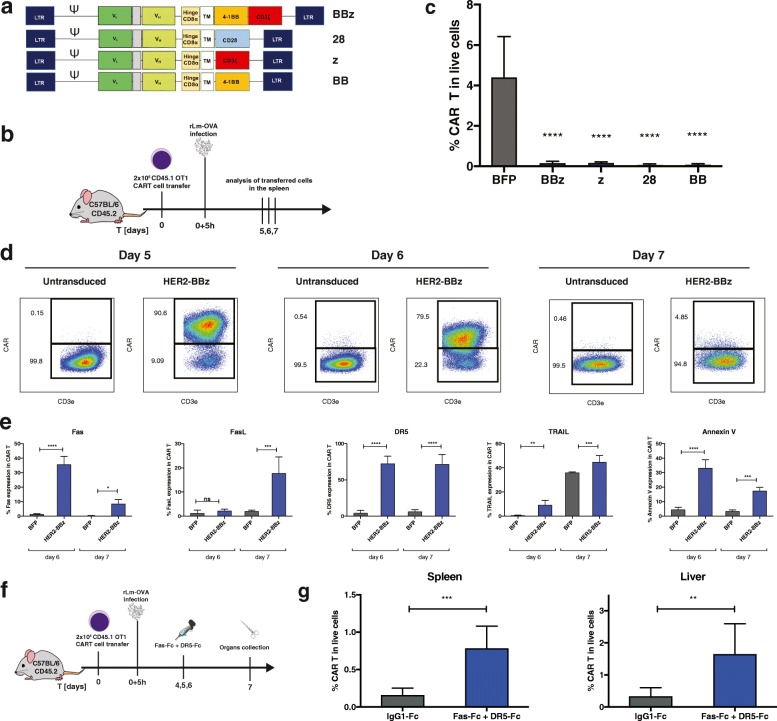
After their initial expansion, HER2-specific CART cells undergo apoptosis. **a** Schemes of tested HER2-CAR configurations. **b** Schematic representation of the experimental protocol **c** Frequencies of BFP or HER2-CART cells bearing various CAR co-stimulatory domains out of total live cells in the spleen at day 7 post rLm-OVA infection. **d** Representative FACS plots of CART cell frequencies of CART cells at day 5, 6 and 7 in the transferred OT-1 population. **e** Fas, FasL, DR5, TRAIL and Annexin V expression and viability staining in BFP or CAR-positive OT-1 T cells at day 6 and 7 post infection. **f** Scheme of the in vivo rescue of CART cells with recombinant Fas-Fc and DR5-Fc recombinant proteins. **g** Percentage of HER2- positive OT-1 T cells at day 7 post infection with rLm-OVA in the spleen and liver of mice that received either the control *h*IgG1 or both Fas-Fc and DR5-Fc. Statistical analysis by One-way ANOVA (**c** and **e**) and by T-test (**g**), *n* = 4 (**c-e**) or 5 (**g**) mice per group. * *P* < 0.05, ** *P* < 0.01, *** *P* < 0.001 and **** *P* < 0.0001

**Fig. 2:**
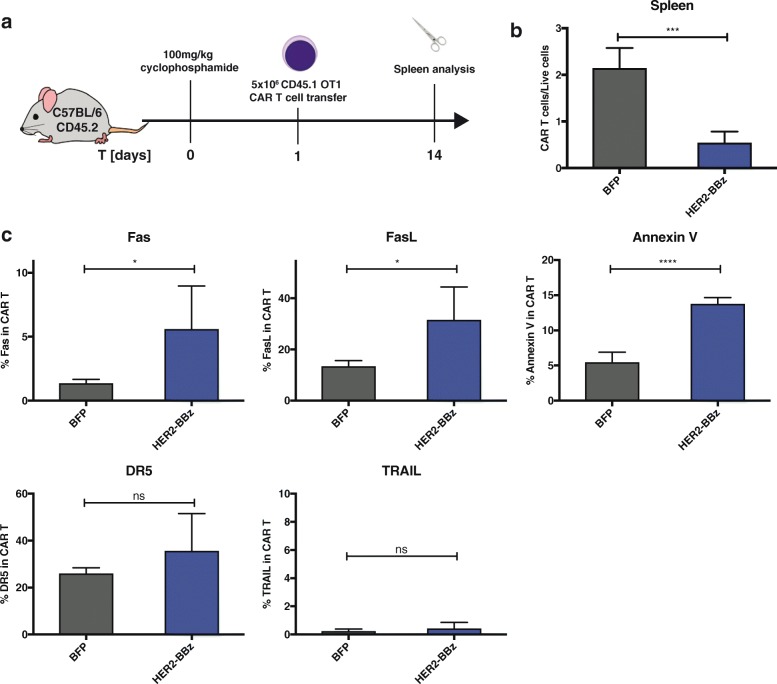
The programmed cell death of CD8 CART cells occurs independently of TCR engagement. **a** Scheme of the experiment involving either BFP or HER2-BBz CAR transduced OT-1 T cells in mice that were conditioned with cyclophosphamide. **b** Frequencies of BFP or HER2-CART cells out of total live cells in the spleen at day 14 post transfer. **c** Fas, FasL, DR5, TRAIL and Annexin V expression in BFP or HER2-CAR OT-1 T cells at day 14 post transfer. Statistical analysis by T-test, *n* = 4 mice per group. * *P* < 0.05, ** *P* < 0.01, *** *P* < 0.001 and **** *P* < 0.0001

**Fig. 3:**
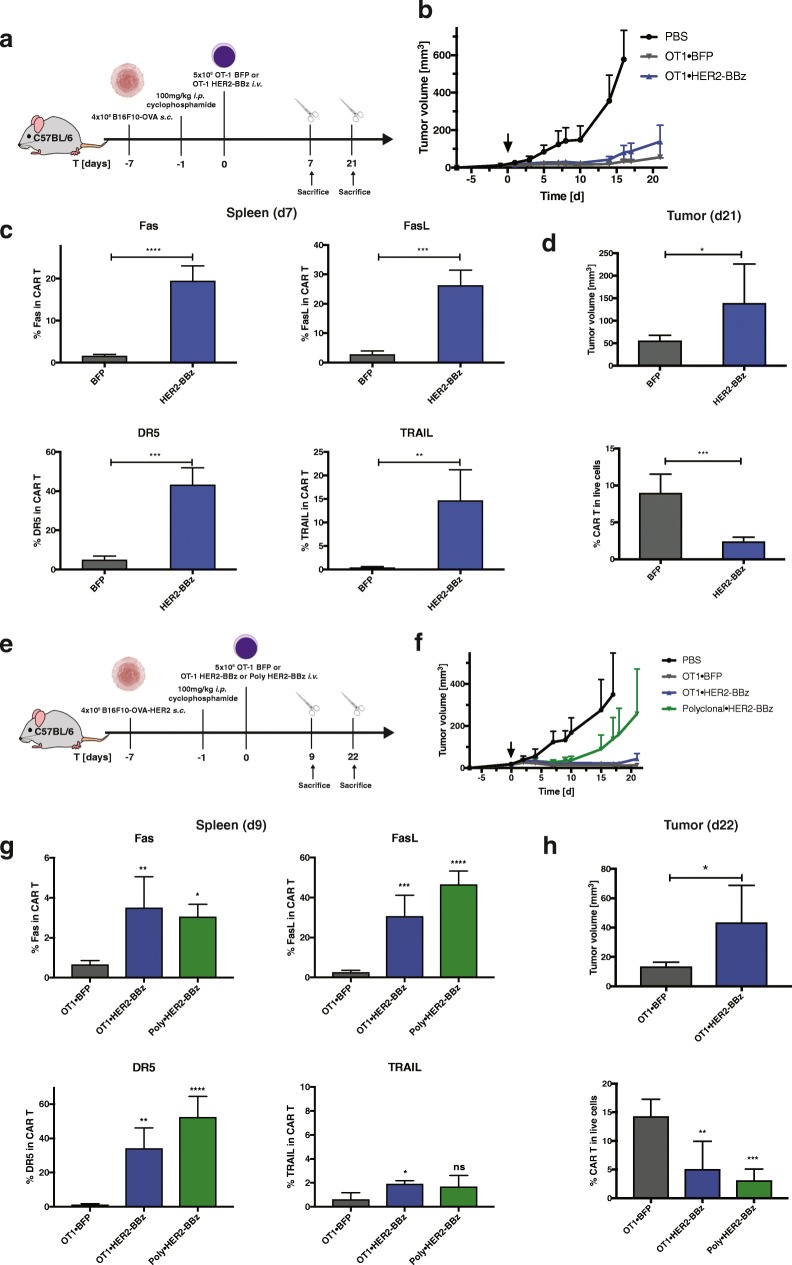
The susceptibility of CART cells to programmed cell death progressively decreases their anti-tumoral capacity. **a** Scheme of the experiment in which mice were engrafted with B16-OVA tumors, conditioned with cyclophosphamide and treated with BFP or HER2-BBz OT-1 CART cells. **b** Evolution of tumor volumes over time. **c** Expression of Fas, FasL, DR5 and TRAIL in BFP or CART cells in the spleen at day 7 post transfer. **d** Tumor volume and percentage of BFP or CART cells in total live cells in the tumors at day 21 post transfer. **e** Scheme of the experiment in which mice were engrafted with B16-OVA-HER2 tumors, conditioned with cyclophosphamide and treated with BFP or HER2-BBz OT-1 or polyclonal CART cells. **f** Evolution of tumor volumes over time. **g** Expression of Fas, FasL, DR5 and TRAIL in BFP or CART cells in the spleen at day 9 post transfer. **h** Tumor volume and percentage of BFP or CART cells in total live cells in the tumors at day 22 post transfer. Statistical analysis by T-test (**c, d, h**) and by One-way ANOVA (**g, h**). Each sample were compared to OT1⋅BFP (**g**), *n* = 10 mice per group. * *P* < 0.05, ** *P* < 0.01, *** *P* < 0.001 and **** *P* < 0.0001

H-2Kb/OVA specific OT-1 T cells were transduced with a human-specific HER2-CAR bearing different combinations of co-stimulatory signaling domains (Fig. 1a), which were validated in vitro (Additional file 1: Figure S1). All CAR configurations were functional except the HER2-CAR with single domain CD28 or 4-1BB, as they lacked the CD3ζ domain. First, we assessed the CART cell response upon in vivo engagement of the resident TCR by cognate antigen on microbial infection. To this aim, HER2-CAR OT-1 T cells were transferred into recipient mice, which were subsequently infected with recombinant *Listeria monocytogenes* bacterium genetically modified to express the chicken ovalbumin sequence (OVA_133–387_), referred thereafter as rLm-OVA (Fig. 1b) [15]. We found that all CART cells, regardless of the CAR construct, had almost completely disappeared at the peak of infection on day 7, in contrast to the large expansion of control-transduced (BFP) T cells (Fig. 1c). Strikingly, even the single co-stimulatory domains CD28 and/or 4-1BB were able to mediate CART cell death following stimulation of the resident TCR, although the CAR itself lacking the CD3ζ domain was not functional (Additional file 1: Figure S1). Longitudinal monitoring of the T cell response at earlier time points showed that HER2-CART cells bearing the co-stimulatory domains CD3ζ and 41BB domains (BBz) expanded efficiently until day 5 post-infection, then declined by day 6 and had mostly disappeared on day 7 (Fig. 1d). The dramatic loss of CART cells was seen in blood, spleen, mesenteric lymph nodes and liver (Additional file 1: Figure S2), and correlated with the up-regulation of Fas, FasL, DR5, TRAIL and Annexin V on days 6 and 7, suggesting their possible deletion via Fas and DR5-mediated AICD (Fig. 1e). The upregulation of cell death markers was seen with all configurations of CAR co-stimulatory domains, as seen by Fas, FasL, DR5, TRAIL and Annexin V up-regulation, albeit with slightly different amplitudes (Additional file 1: Figure S3). The Fas and DR5 signaling pathways were necessary for TCR-induced CART cell apoptosis, as shown by the significant rescue of CART cells in spleen and liver upon systemic treatment with the cocktail of recombinant Fas-Fc and DR5-Fc proteins (Fig. 1f). To further assess if TCR triggering was required for CART cell apoptosis, we monitored the survival of 5 × 10^6^ HER2-CARBBz or BFP OT-1 T cells transferred in naïve B6 mice, which were lymphodepleted with cyclophosphamide to allow engraftment of T cells in the absence of antigen stimulation. Strikingly, CAR OT-1 T cells were also prone to PCD in the absence of the OVA antigen and CAR activation, as seen by their reduced frequencies at day 14 post CART cell transfer associated with the upregulation of Fas, FasL, and Annexin V (Fig. 2). Of note, DR5 and TRAIL were not upregulated on CART cells in the absence of TCR triggering, suggesting that additional death signals might be induced upon concomitant TCR and/or CAR activation. The susceptibility of CART cells to PCD was not peculiar to the HER2-CAR, as OT-1 T cells transduced with a CEA-CAR also upregulated Fas and FasL and underwent subsequent cell death upon rLm*-*OVA infection in the absence of CAR activation (Additional file 1: Figure S4). In view of the clinical success of CART cells in cancer immunotherapy, we evaluated whether CART cell susceptibility to apoptosis would compromise their antitumor activity against tumors expressing the TCR and/or CAR antigen (Fig. 3a and e). When 5 × 10^**6**^ BFP or HER2-CAR OT-1 T cells were transferred in cyclophosphamide pre-conditioned mice bearing B16-OVA tumors, the TCR-mediated antitumor effects were similar for the first three weeks post tumor graft (Fig. 3b). However, a significant upregulation of cell death markers was already detected at day 7 post ACT on spleen (Fig. 3c) and tumor-infiltrating CART cells (data not shown). As a consequence, frequencies and tumor control of CART cells progressively decreased, and on day 28 post tumor graft, tumor volumes were significantly larger than in mice transferred with control BFP-OT-1 cells, which was associated with decreased tumor infiltration of OT-1 CART cells (Fig. 3d). Importantly, when engaging OT-1 CART cells via both the OT-1 TCR and the HER2-CAR against B16-OVA/HER2 tumors (Fig. 3e), there was no sign of additive antitumor effect, while the HER2-CAR was able to mediate significant antitumor effect when transduced in naïve CD8 T cells bearing polyclonal TCRs (Fig. 3f). Similar to what was observed in the B16-OVA model, Fas, FasL, DR5 and TRAIL were significantly upregulated, and to a similar extent on spleen OT-1 and polyclonal CD8 CART cells on day 9 post ACT (Fig. 3g). Accordingly, on day 28 post tumor graft, tumor volumes were significantly larger in mice transferred with CART cells, which correlated with reduced tumor infiltration of CART cells, whether the CAR was transduced in OT-1 or polyclonal CD8 T cells (Fig. 3h).

Overall, we found that CART cells are intrinsically prone to Fas- and DR5-mediated programmed cell death, as shown by their partial rescue upon blockade of these death signaling pathways. The progressive loss of CART cells in tumor bearing mice, did not hinder TCR or CAR-mediated antitumor effects, but prevented their long-term therapeutic effects, as well as the additive antitumor activity via combined TCR and CAR tumor targeting. Strikingly, the susceptibility of CART cells to cell death occurred with all tested CAR configurations even those with a single CD28 and/or 4-1BB CAR domain. These results were particularly unexpected as CD28 and 4-1BB pathways have been rather associated with T cell persistence and resistance to exhaustion [16, 17]. However, depending on the context, both co-stimulatory domains might also be involved either directly or indirectly in the induction of T cell apoptosis [18, 19]. We can speculate that the efficient transduction of CARs leads to a supra-physiological expression levels of CD3ζ, CD28 or 4-1BB domains, as compared to their endogenous expression, which may result in some tonic signaling upon TCR/MHC cognate interaction. The slow kinetic of CART cell death shown in the homeostatic or tumor settings, versus the fast CART cell loss in the rLm-OVA model, suggests that the intensity of TCR triggering and the inflammatory milieu are playing a role. For instance, CD28 has been shown to recruit Lck [20], which could cooperate and increase TCR signaling resulting in increased NF-κB and induction of Fas/FasL expression. While the role of enhanced CD28 signaling seems rather indirect, 4-1BB signaling has been directly associated with Fas/FasL-dependent apoptosis of T cells in defined contexts [19], in particular, in the presence of high CD19-CAR expression containing a 4-1BB domain [18]. Therefore, it will be important to determine whether high expression levels of CAR-associated co-stimulatory domains generate a weak but constitutive activation signal in T cells, which may lead to a low noise level of NFκB activation sufficient to induce Fas/FasL upregulation. Moreover, the constitutive resident TCR engagement by low affinity self MHC/peptide ligands [21], may contribute to an aberrant CART cell activation.

Interestingly, another study has recently reported TCR-mediated CART cell apoptosis [22], using an in vivo model involving the rejection of CD19-CART cells when expressing a TCR specific for the HY-male antigen. Consistent with our study in acute infection models, deletion of female HY-specific CD19-CART cells was observed upon their transfer in HY^+^ male hosts. Intriguingly, T cell attrition in that model was more severe in CD8 than in CD4 T cells. As a consequence, CD8 CART cells failed to control B cell lymphoma tumors, while CD4 T cells retained antitumor effect but were ultimately also deleted. As the TCR antigen was presented by all male cells, the loss of HY-specific CD19-CART cells was likely more dramatic than in our study in which TCR triggering was restricted to OVA-expressing tumor cells, which likely allowed longer survival or CART cells and tumor inhibition. Another supportive indirect evidence of TCR dysfunction in CART cells was reported recently in clinical trials involving the transfer of allogeneic CD19-CART cells in pediatric leukemic patients [23, 24]. Strikingly, a significant decreased GvHD was observed when allogeneic donor cells were expressing a CAR. The same authors confirmed a diminished GvHD in a subsequent pre-clinical study, and showed that alloreactive CART cells were characterized by enhanced stimulation associated with progressive loss of effector function, proliferation and clonal deletion [25]. In view of these results, we hypothesize that the persistence of CART cells bearing an alloreactive TCR was decreased due to alloantigen-mediated activation and accelerated cell death, which was instrumental for the decreased GvHD. In that study, decreased GvHD was selectively associated with CD28-bearing CAR but not for 4-1BB or first generation’s CARs. Instead, in our models, the apoptosis of CART cells took place regardless of the number and type of co-stimulatory domains, and independently of TCR and CAR activation. We therefore speculate that the intrinsic susceptibility of CART cells to programed cell death might be further enhanced upon TCR and/or CAR activation. Of note, a recent study has reported that the CRISPR/Cas9-mediated targeting of the CAR to the TCR locus prevents CAR tonic signaling and delays CART cell exhaustion, which results in enhanced tumor rejection [26]. Although this approach would not allow the combined TCR and CAR activation, it would be very interesting to see if such TCR-deficient CART cells would have decreased susceptibility to PCD and therefore better persistence.

Many aspects of CART cell persistence and survival remain to be studied both in pre-clinical and clinical situations. However, these recent data including ours have revealed a so far poorly appreciated susceptibility of CART cells to PCD. Our study does not challenge the success of CART cells in cancer patients, but demonstrates the need to ameliorate the long term persistence of CART allowing combined TCR and CAR activation, in particular for the treatment of solid tumors. In this context, alternative strategies may be developed, which include the careful control of the dose of CAR expressed, the CAR gene targeting in the TCR gene locus, the potential use of CD4 instead of CD8 T cells as CART cell recipient, or possibly the CART cell intrinsic blockade of apoptotic pathways.

## Methods

### Mice

Female mice C57BL/6 J (B6) six to eight weeks old (Envigo, Gannat, France) were maintained in specific pathogen-free conditions. All animal experiments were conducted according to institutional guidelines and under the authorization VD1605 delivered by the Swiss veterinary department.

### Retroviral constructs

BFP fluorescent protein, HER2 and CEA-specific CARs were cloned in the MSGV retroviral transfer vector [27] under the control of the 5′ LTR promoter. For the HER2-CAR, the plasmid pIG6-4D5, containing the scFv fragment derived from the human-specific anti-HER2 murine antibody 4D5, was used as template [28] (kind gift from A. Pluckthun, University of Zurich, Switzerland). For the CEA-CAR, the scFv MFE23 [29] (kindly provided by R.H. Begent) was used. The single chain antibody fragment was fused to the CD8α hinge and transmembrane domains followed by mouse intracellular TCR signaling endodomains.

### Retrovirus preparation

For each retroviral preparation, 8 × 10^6^ Phoenix ECO cells (ATCC, CRL-3214) were plated in a T150 tissue culture flask in RPMI medium supplemented with 10% FCS, 10 mM HEPES and 50 U/ml Penicillin-Streptomycin. On the next day, cells were transfected with 21 μg of the retroviral construct with Turbofect transfection reagent (Thermo Fischer Scientific), according to the manufacturer protocol. The medium was changed daily and collected at 48 h and 72 h post transfection. 48 h and 72 h virus supernatants were pooled and sedimented at 22000rcf for 2 h at 4 °C. Finally, retrovirus pellets were resuspended in 2 ml of full RPMI medium and divided in 8 aliquots of 250 μl each, which were snap-frozen on dry ice and stored at − 80 °C.

### Mouse CD8 T cells transduction

Spleens from CD45.1xOT1 or CD45.1xP14 transgenic mice were smashed on a 40 μm cell strainer. CD8 T cells were purified using the EasySep™ Mouse CD8^+^ T Cell Isolation Kit (StemCell) according to the manufacturer protocol. 0.5 × 10^6^ CD8 T cells were plated in 48well plates in 0.5 ml of complete RPMI 1640 medium supplemented with 10% FCS, antibiotics and 50 IU/ml of recombinant human IL-2. Mouse T-cells were activated with Activator CD3/CD28 Dynabeads (Gibco) at a ratio of 2 beads per cell. Retroviral infection was conducted at 37 °C for 24 h. Untreated 48well plates were coated for 24 h with 20 μg/ml of recombinant human fibronectin (Takara Clontech) at 4 °C, followed by PBS 2% BSA for 30 min at RT and finally washed with PBS. One aliquot of concentrated retroviruses was plated in each fibronectin-coated 48well plates and centrifuged for 90 min at 2000rcf and 32 °C. Then, 0.5 × 10^6^ of 24 h-activated CD8 T cells were added on top of the viruses and spun for 10 min at 400rcf and 32 °C. Medium was renewed daily and cell density was kept below 2 × 10^6^ cells/ml. On day 3, the medium was supplemented with 10 IU/ml recombinant human IL-2, 10 ng/ml recombinant human IL-7 and 10 ng/ml recombinant human IL-15. From day 5 post activation, the cells were fed with only IL-7 and IL-15.

### rLm-OVA infection model

BFP or CART cells were harvested and CD3/CD28 Dynabeads were removed. Cells were counted and washed 3 times in PBS. 0.2 × 10^6^ cells were resuspended in 200 μl of plain RPMI medium and injected in the mouse tail vein.

### rLm-OVA infection

2000 *cfu* were injected *i.v.* in 200 μl of PBS.

### Therapeutic tumor model

C57BL/6 mice were engrafted subcutaneously with 4 × 10^5^ B16F10 tumors modified to express the OVA antigen with or without HER2. Six days later, mice were lymphodepleted with 100 mg/kg cyclophosphamide (Sigma Aldrich, C7397) injected *i.p*., and homogeneous groups were constituted with regard to tumor volume. T cells (5 × 10^6^) were adoptively transferred *i.v*. on the next day. Kinetic of tumor growth was monitored every two days with an electronic caliper. CART cell analysis in the spleens was performed as described previously. Tumors were collected and separated from skin. Single cell suspensions were obtained with the Mouse Tumor Dissociation Kit (Miltenyi, 130–096-730) according to the manufacturer protocol.

### Sample preparation, flow cytometry staining and acquisition

Spleens were collected at indicated days post rLm-OVA, and smashed on 40 μm cell strainers to obtain single cell suspensions. Red blood cells were lysed using a RBC Lysis Solution (Qiagen). Livers were smashed using a tea strainer. Large debris were discarded with a low speed centrifugation. Then, lymphocytes were enriched by density separation with two phases of 40 and 70% Percoll (GE Healthcare, GE17–0891-01). Single cell suspension from lymph nodes were obtained by smashing the organs on a 40 μM strainer. Blood samples were treated with RBC Lysis solution. Prior to labelling, Fc receptors were blocked using 2.4G2 supernatant. Cells were labelled with Zombie Yellow™ Fixable Viability Kit (Biolegend) to discriminate dead cells. HER2-specific CARs were labelled with HER2-Fc (Sino Biological) at 5 μg/ml on ice for 20 min and then washed 3 times with PBS, 2% FCS, 2.5 mM EDTA (facs buffer). Next, cells were labelled with Brilliant Violet 421 anti-human IgG Fc Antibody (BioLegend) at 2 μg/ml for 20 min on ice and washed again 3 times. The CEA-specific CAR was instead stained with Fluorescein AffiniPure Goat Anti-Mouse IgG, F(ab’)_2_ (Jackson ImmunoResearch) at 1 μg/ml on ice for 20 min and then washed 3 times. After CAR staining, transduced cells were labelled with CD3-Alexa Fluor 700 at 10 μg/ml (BioLegend, 100,215), CD8α-PE/TexasRed at 1/500 dilution (Thermo Fischer Scientific, MCD0817), CD45.1-APC/eFluor 780 at 2 μg/ml (eBioScience, 47–0453-82), CD45.2-PerCP/Cy5.5 at 2 μg/ml (BioLegend, 109,827), Fas-APC at 2 μg/ml (BioLegend, 152,603) and FasL-Biotin at 5 μg/ml (BioLegend, 106,603). Cells were then washed once and labelled with Streptavidin-PE/Cy7 at 0.4 μg/ml (BioLegend, 405,206). Finally, cells were washed again and stained with Annexin V-PE (BioLegend, 640,907) according to the manufacturer protocol, and acquired on a BD LSR2 flow cytometer.

### In vitro cytokine release assay for CART cells

0.2 × 10^6^ OT-1 CART cells were plated in flat-bottom 96well plates with either 0.1 × 10^6^ B16, B16-OVA or B16-HER2 tumor cells. 30 min later, the Monensin and Brefeldin A-containing reagents Golgi Stop and Golgi Plug (Becton Dickinson) were added at the final concentration of 1:1000. Cells were further incubated for 4 h prior to staining for flow cytometry. Cells were labelled for viability and with HER2-Fc as described, followed by surface-staining with anti-human IgG Fc-FITC (BioLegend, 409,309), CD3ε-Alexa Fluor 700 at 10 μg/ml (BioLegend, 100,215) and CD8α-Brilliant Violet 650 at 0.5 μg/ml (BioLegend, 100,741). To stain for intracellular cytokines, cells were fixed and permeabilized (Biolegend) according to the manufacturer protocol, before being stained with the antibodies IFNγ-PerCP/Cy5.5 at 0.5 μg/ml (Biolegend, 505,821) and TNFα-Pacific Blue at 1 μg/ml (Biolegend, 506,318) diluted in permeabilization buffer (Biolegend) for 30 min on ice. Cells were finally washed twice in permabilization buffer and resuspended in FACS buffer prior to acquisition.

### Fas and DR5 blockade

Human Fas-Fc was obtained from Adipogen (AG-40B-0082-C050), and human DR5-Fc and control IgG1-Fc portion was produced at the protein production core facility at EPFL, Lausanne. Specificities of human Fas-Fc and DR5-Fc for blocking their murine orthologs have been previously described [30, 31]. The biological activity of DR5-Fc was compared to a reference sample through its ability to inhibit TRAIL-mediated apoptosis of Jurkat cells. BFP or CAR OT-1 T cells were transferred in C57BL/6 mice, which were subsequently infected with rLm-OVA as described previously. Based on the in vitro testing, 200 μg of Fas-Fc and 500 μg of DR5-Fc or human IgG1 control, were injected *i.v.* on days 4, 5 and 6 post-infection.

### Statistical analyses

Statistical analyses were performed with Graphpad Prism 7. Normally distributed data that include two groups were compared using Two-tailed unpaired T tests. Statistical significance was reached with *P* < 0.05. Normally distributed data with more than two groups were compared using One-way or Two-way ANOVA tests. Multiple comparisons were corrected using Tukey and Sidak tests, respectively. Normality was tested with a Shapiro-Wilk test. On the plots, data represent mean ± SD.

## Additional file

Additional file 1: Supplementary Figures 1-3. (PDF 18433 kb)

## Abbreviations

ACT: Adoptive Cell Transfer
AICD: Activation Induced Cell Death
BFP: Blue Fluorescent Protein
CART: Chimeric antigen receptor-transduced T cells
CEA: Carcinoembryonic antigen 2
GvHD: Graft-versus-host disease
HER2: Human epidermal growth factor receptor
PCD: Programmed Cell Death
rLm-OVA: recombinant *Listeria monocytogenes* expressing OVA_134–387_
TCR: T cell-receptor
