# Correction to: CART cells are prone to Fas- and DR5-mediated cell death

**DOI:** 10.1186/s40425-018-0410-2

**Published:** 2018-09-25

**Authors:** Benjamin O. Tschumi, Nina Dumauthioz, Bastien Marti, Lianjun Zhang, Evripidis Lanitis, Melita Irving, Pascal Schneider, Jean-Pierre Mach, George Coukos, Pedro Romero, Alena Donda

**Affiliations:** 1 Department of Fundamental Oncology, Translational Tumor Immunology Group, Lausanne, Switzerland; 2 Department of Oncology, University Hospital of Lausanne, Lausanne, Switzerland; 3 Ludwig Institute for Cancer Research, Lausanne Branch, Lausanne, Switzerland; 4 Department of Biochemistry, Faculty of Biology and Medicine, University of Lausanne, Lausanne, Switzerland

## Correction

After publication of this article [1], it was noticed that 3 authors were missed from the author list. These authors are Evripidis Lanitis, Melita Irving and George Coukos. The correct author list can be seen above and updated Competing Interests, Authors’ Contributions and Acknowledgements can be seen below.
