# Supplementary material for: CART cells are prone to Fas- and DR5-mediated cell death
Source: J Immunother Cancer. 2018 Jul 13;6:71. doi: 10.1186/s40425-018-0385-z (PMC6045821; doi:10.1186/s40425-018-0385-z)
Supplement: Supplementary file 1 — Supplementary Figures 1-3. (PDF 18433 kb) [file 40425_2018_385_MOESM1_ESM.pdf]

Figure S1

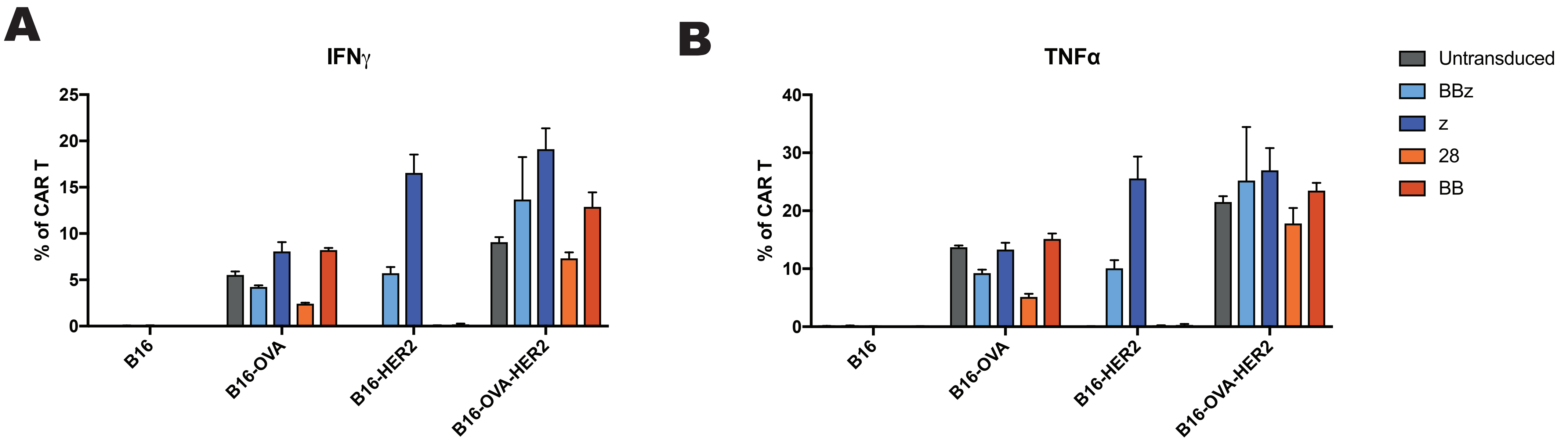

In vitro TCR and CAR functionality in OT-1 T cells transduced with BBz, 28, BB or z HER2-CAR configurations. Untransduced or HER2-CAR OT-1 T cells were co-cultured with B16, B16-OVA, B16-HER2 or B16-OVA-HER2 for 4h and stained for intracellular **(A)** IFNγ **(B)** TNF-α accumulation.

Figure S2

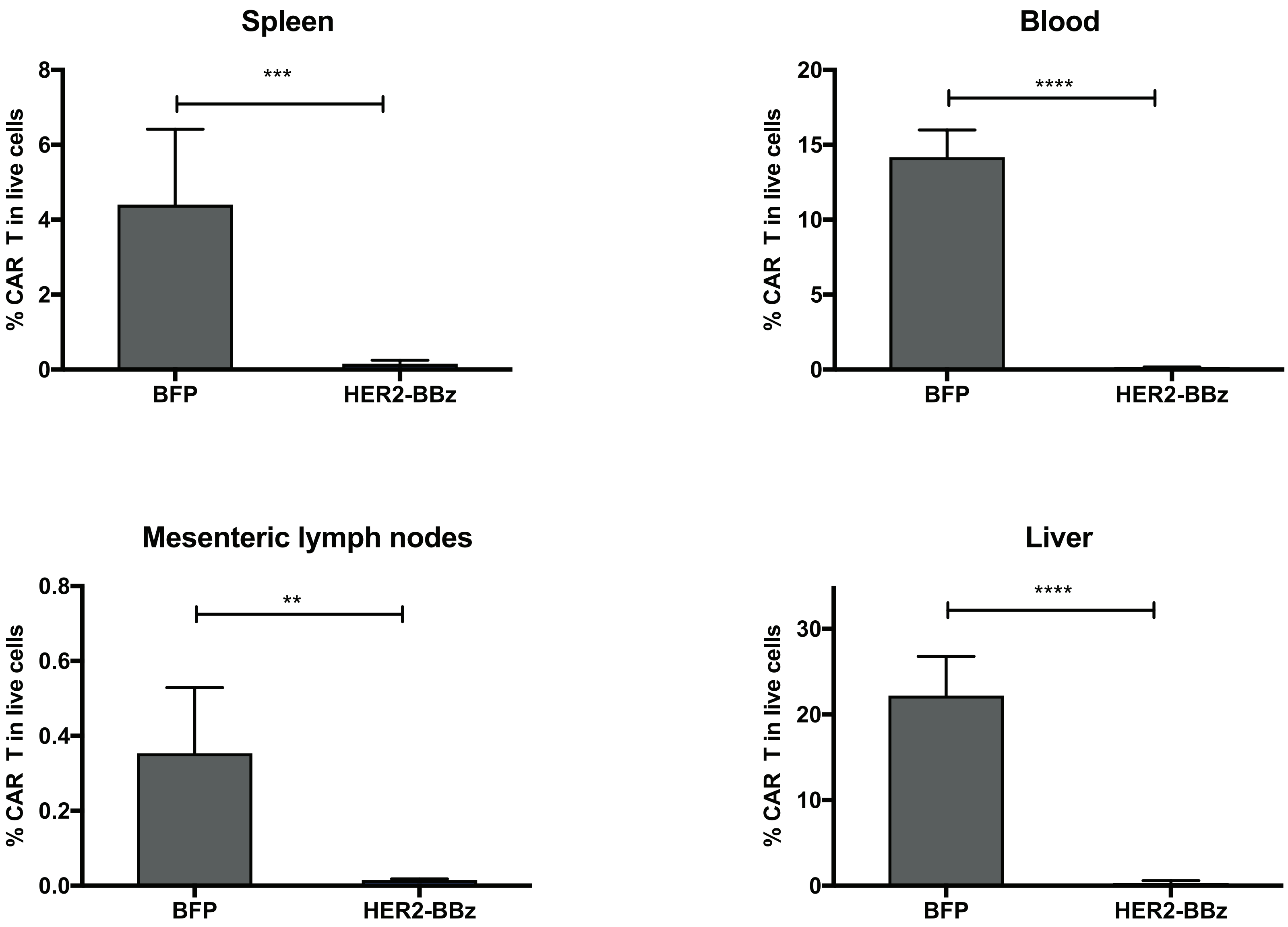

The deletion of CAR T cells occurs in multiple organs. BFP or HER2-BBz OT-1 CD45.1 CD8 T cells were transferred in CD45.2 mice that were subsequently infected with rLm-OVA. Graph shows frequencies of BFP or CAR OT1 T cells as percent of live cells at day 6 (blood) and 7 (organs) post rLm-OVA. Statistical analysis by T-test, n=5 mice per group. * $P < 0.05$, ** $P < 0.01$, *** $P < 0.001$ and **** $P < 0.0001$.

Figure S3

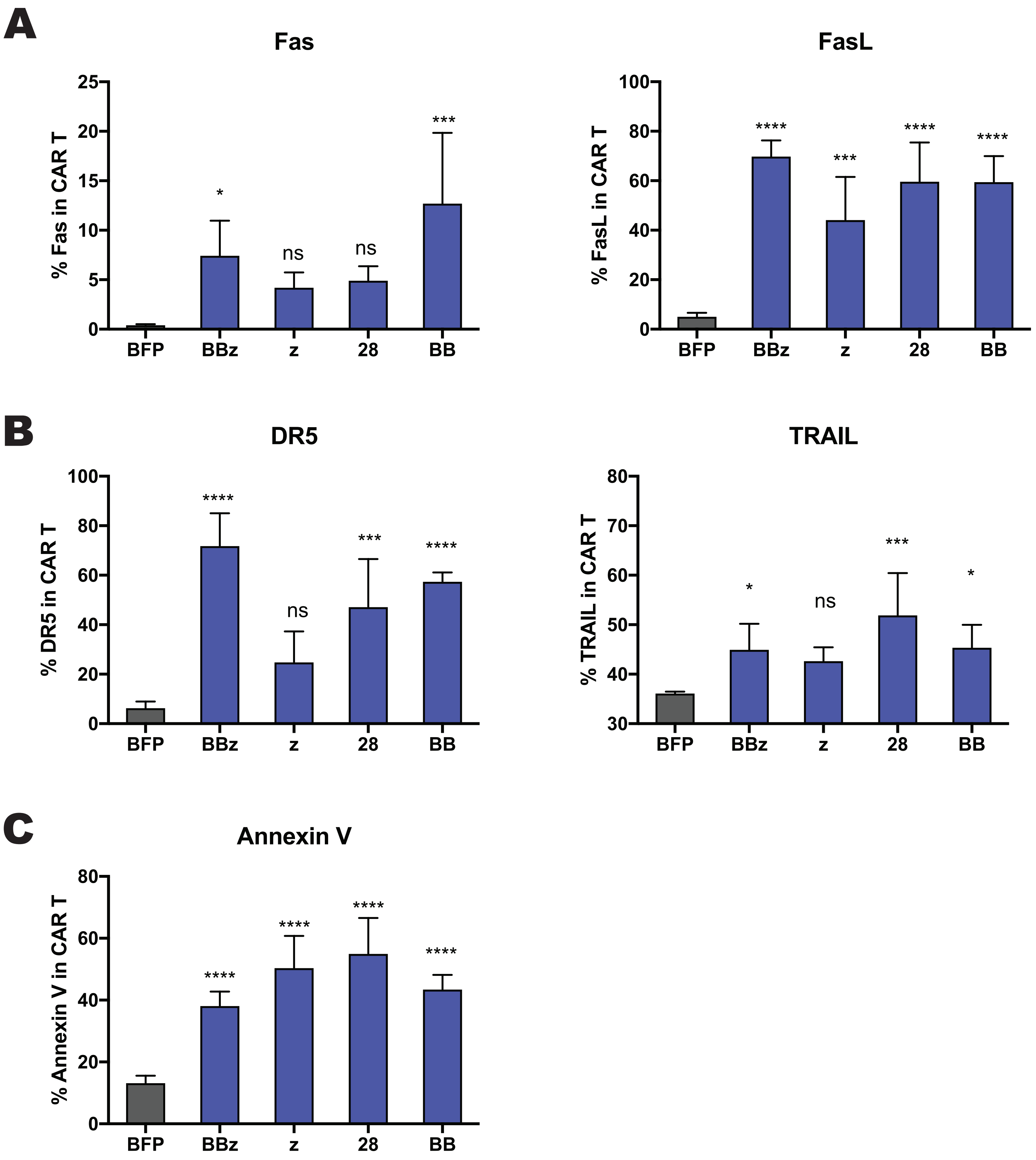

The different CAR configurations induce Fas, FasL, DR5, TRAIL and Annexin V expression to various extent. (A) Fas, FasL, **(B)** DR5, TRAIL and **(C)** Annexin V expression in BFP or HER2 CAR-positive OT-1 T cells at day 7 post rLm-OVA infection in the spleen. Statistical analysis by one-way ANOVA, each sample was compared to BFP, n=4 mice per group. * $P < 0.05$, ** $P < 0.01$, *** $P < 0.001$ and **** $P < 0.0001$.

Figure S4

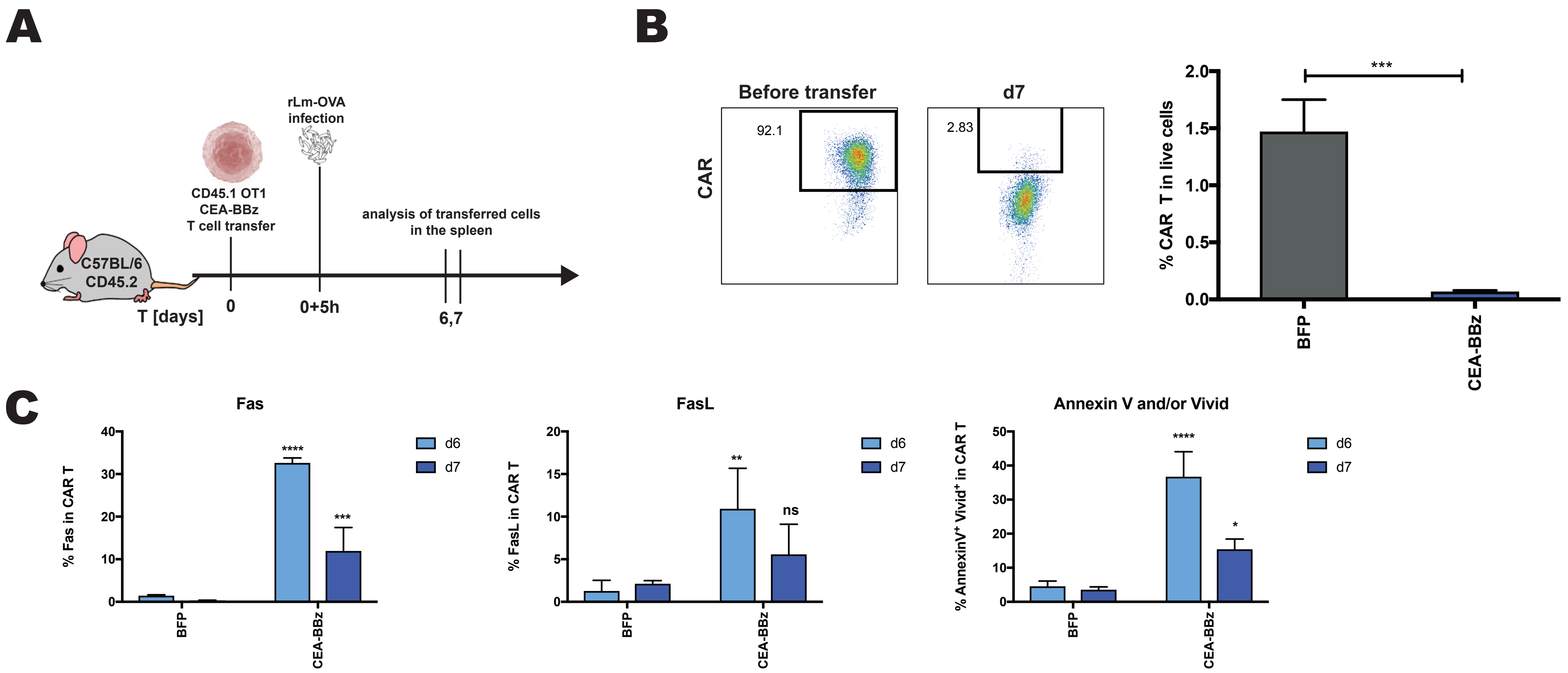

The apoptosis of CD8 CAR T cells is independent of the CAR specificity. (A) Scheme of the experiment involving CEA-CAR OT-1 T cells transferred in mice infected with rLm-ova. **(B)** Dot plots showing frequencies of CEA-CAR OT-1 cells before transfer and at day 7 post rLm-OVA infection. Graph shows frequencies of BFP or CAR OT1 T cells as percent of live cells at day 7 post rLm-OVA. **(C)** Fas, FasL and Annexin V and viability staining in BFP or CAR-positive transferred OT-1 T cells at day 6 and 7 post infection. Statistical analysis by T-test (B) or two-way ANOVA (C), each sample was compared to BFP, n=4 mice per group. * P < 0.05, ** P < 0.01, *** P < 0.001 and **** P < 0.0001.
